# Salvaging the Constricted or Lost Umbilicus After Abdominoplasty With Early Mercedes Release, Silver Nitrate, and Foam Earplug Stenting: Stairway to Heaven

**DOI:** 10.7759/cureus.111088

**Published:** 2026-06-18

**Authors:** Quinton L Carr, Colton H Connor, Claire Tinkler, Alyssa Nguyen, Mitchell Peake, Dexter Weeks, Joshua MacDavid, Bradon J Wilhelmi

**Affiliations:** 1 Plastic and Reconstructive Surgery, University of Louisville School of Medicine, Louisville, USA; 2 Plastic and Reconstructive Surgery, University of Louisville Hospital, Louisville, USA; 3 Plastic Surgery, University of Nevada Las Vegas, Las Vegas, USA

**Keywords:** abdominoplasty, aesthetic surgery, plastic and reconstructive surgery, umbilical reconstruction, umbilicoplasty

## Abstract

Umbilical stenosis is an underrecognized complication following abdominoplasty, driven by cicatricial contracture, myofibroblast-mediated centripetal tension, and stalk ischemia. As the central aesthetic focal point of the abdomen, umbilical malposition or deformity is among the most frequent contributors to postoperative dissatisfaction. We present a 40-year-old female who developed umbilical stenosis 9 months following abdominoplasty with native stalk preservation, managed successfully with a minimally invasive outpatient protocol. A Mercedes-pattern incision was employed to release circumferential scar tension, followed by topical silver nitrate application to attenuate granulation tissue formation and modulate the wound-healing cascade, and temporary foam earplug stenting to maintain aperture patency during scar maturation. At the three-month follow-up appointment, marked improvement in umbilical depth, contour, symmetry, and patency was demonstrated without recurrent stenosis. This combined technique offers a simple, reproducible, low-cost approach to preserving umbilical aesthetics following abdominal body contouring procedures.

## Introduction

Abdominal body contouring is frequently performed in the United States, often sought by patients wishing to address excess abdominal skin, persistent adiposity, weakened abdominopelvic musculature, or the appearance of stretch marks [1]. These concerns most commonly follow significant weight loss, pregnancy, or aging [2,3]. While surgical indications may be functional or aesthetic, patient-reported outcomes are largely driven by aesthetic appearance. Among the most frequent contributors to postoperative dissatisfaction are the surgical scar and the appearance of the umbilicus, emphasizing the importance of an aesthetically balanced result [4-6].

The umbilicus holds profound cultural, psychological, and aesthetic significance across civilizations, from the acupuncture meridians in ancient Chinese medicine to the modern Western aesthetic ideal prominently displayed in a two-piece bathing suit. Ancient East Asian cultures regarded the umbilicus as spiritually consequential, transcending its cosmetic role. In Japanese tradition, the umbilicus was considered the seat of one's vital life force, while Chinese medicine viewed it as a sacred spiritual gateway--the center of heaven itself--perhaps serving as a "stairway to heaven.” This reverence extended to clinical practice, with the umbilical region used therapeutically to treat a range of systemic ailments, a testament to its perceived position at the convergence of the body's most vital forces [7].

Consistent with this significance, modern patients prefer to maintain an umbilicus after surgery, whether preserved or reconstructed [4,8]. Although functionally vestigial in adults, its absence can be distressing for both aesthetic and reconstructive patients [4]. Notably, postoperative satisfaction is determined less by native versus reconstructed status than by the naturalness of the umbilicus’s appearance, position, and contour [4,8]. Collectively, these findings reinforce that dissatisfaction is most strongly linked to unfavorable appearance or malposition rather than absence alone, and that preservation of a naturally contoured umbilicus remains a paramount objective in abdominal body contouring [5,6].

## Case presentation

**Figure 1:**
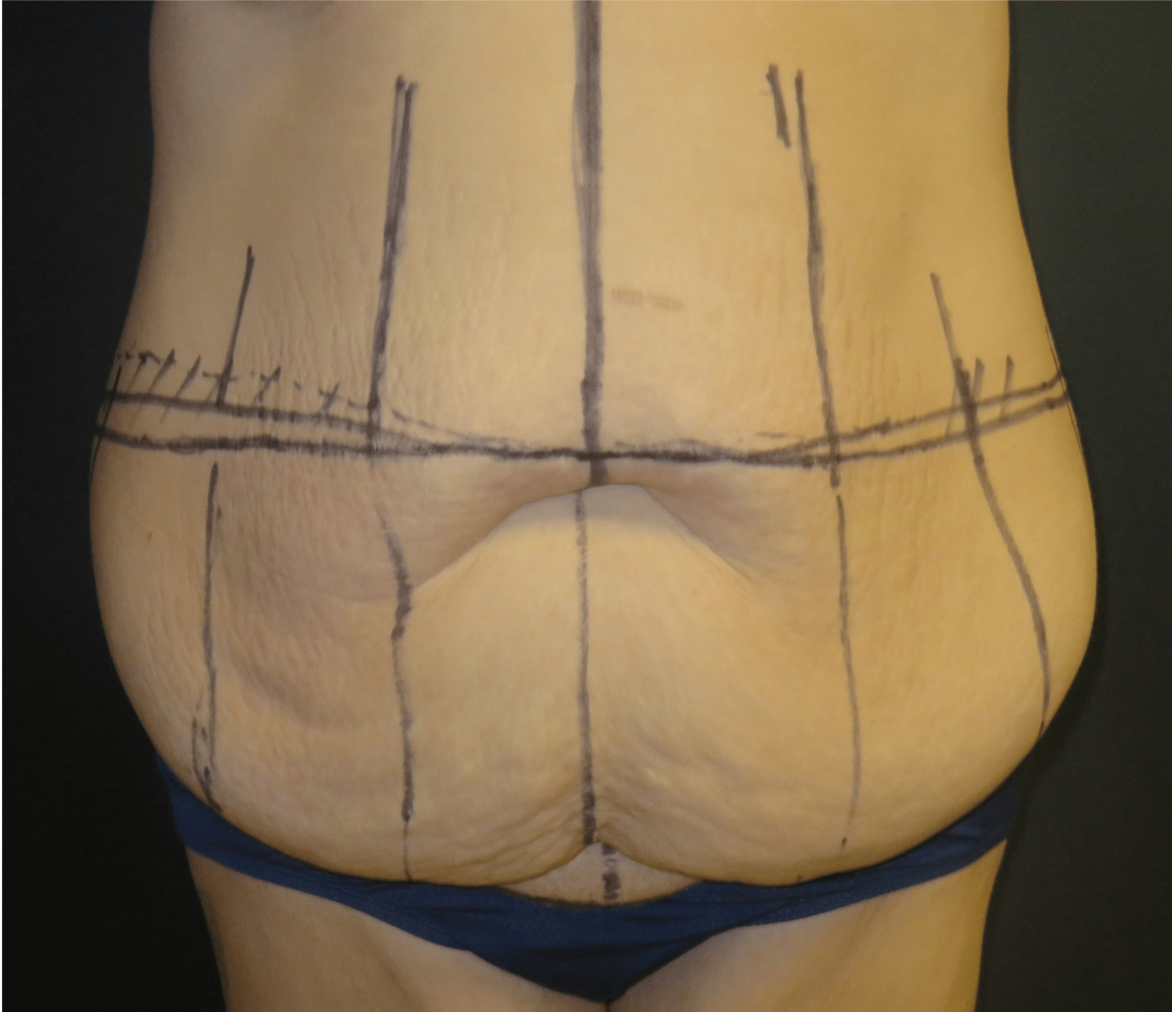
Pannus at initial presentation Preoperative appearance of the abdomen prior to abdominoplasty

**Figure 2:**
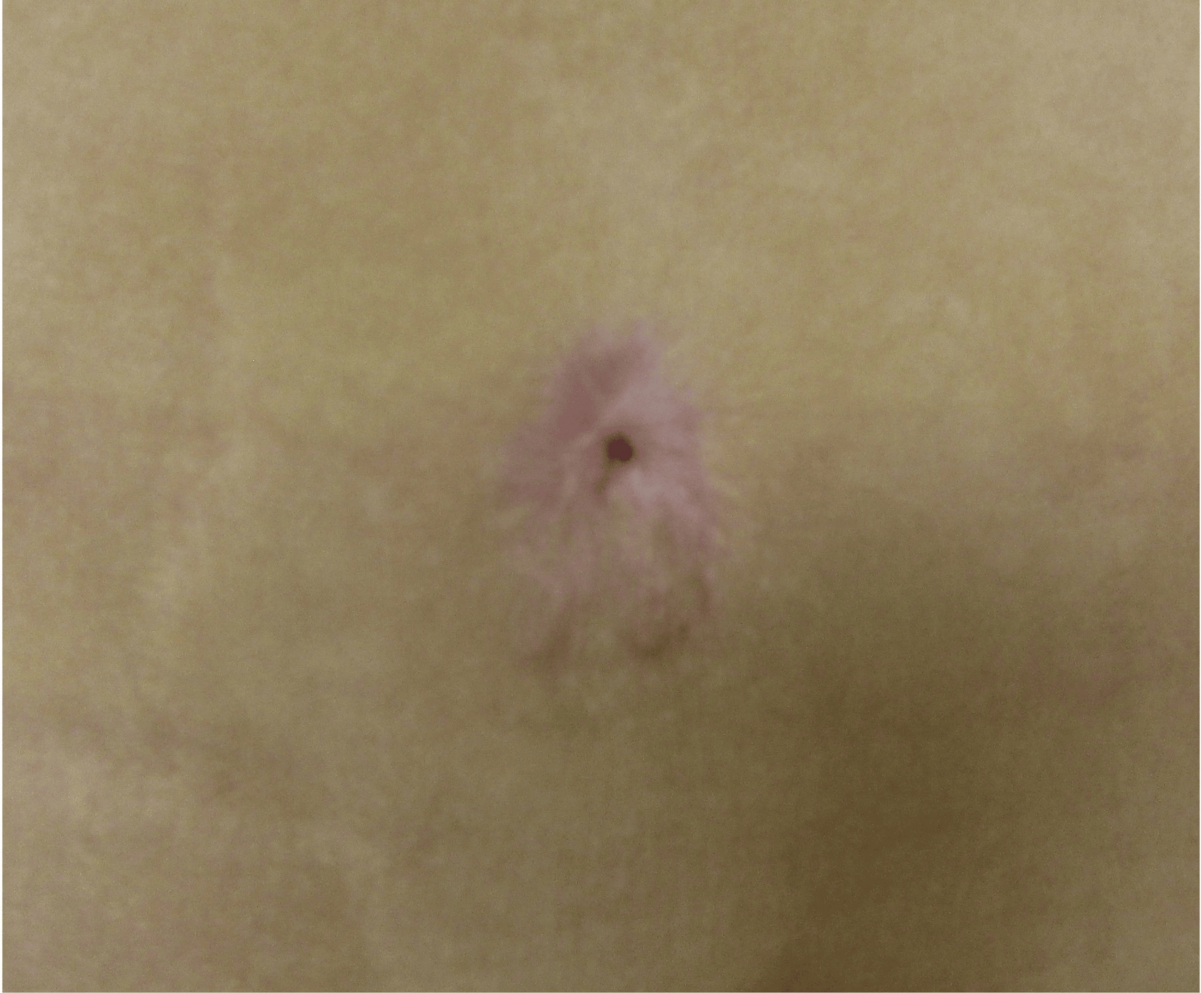
Umbilical stenosis Postoperative umbilical stenosis nine months post-abdominoplasty demonstrating circumferential cicatricial contracture, reduced aperture patency, and a flattened, recessed contour

A 40-year-old female with a history of massive weight loss (approximately 130 lbs) following bariatric surgery presented with chronic infrapannicular intertrigo and lower back pain refractory to conservative medical management (Figure 1), prompting evaluation and referral for abdominoplasty. The patient elected to undergo the procedure with umbilical preservation on its native stalk. The initial postoperative course was uneventful. However, nine months postoperatively, she developed progressive umbilical stenosis with circumferential cicatricial contracture and gradual reduction of the umbilical aperture, resulting in a flattened and constricted contour detracting from an otherwise satisfactory aesthetic result. An image of the stenotic umbilicus is displayed in Figure 2. Aside from bariatric surgery, the patient's past surgical history was otherwise unremarkable for abdominal operations. She had no known risk factors for tissue ischemia or umbilical stenosis beyond an elevated BMI in the setting of prior obesity. Though vascular compromise and cicatricial contracture are both recognized contributors to umbilical stenosis, it was not possible to definitively determine the primary cause of stenosis in this patient.

**Figure 3:**
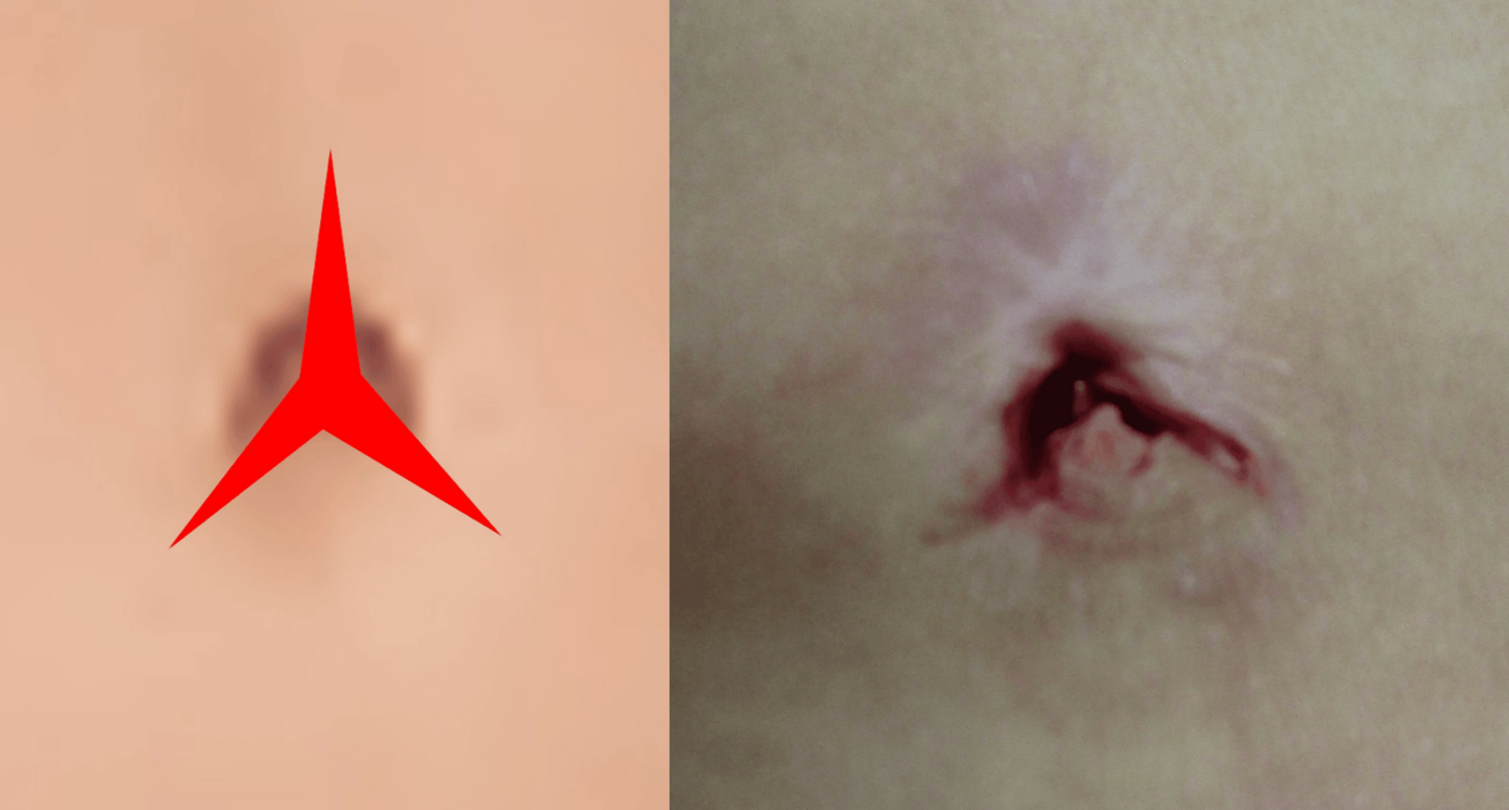
Mercedes-pattern incision design and procedural appearance The schematic (left) illustrates the three radial limbs extending from the umbilical aperture to release circumferential scar tension while preserving native umbilical tissue, corresponding to the photograph (right) demonstrating the incision as applied to the stenotic umbilicus during the bedside procedure.

A minimally invasive outpatient corrective technique was employed to restore cosmesis. A Mercedes-pattern incision was utilized to release circumferential scar tension and re-establish aperture patency while preserving the native umbilical stalk (Figure 3). 

**Figure 4:**
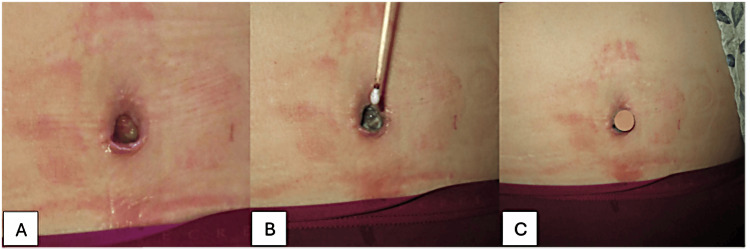
Silver nitrate reapplication process: one-month follow-up Umbilical appearance at the one-month follow-up demonstrating marked improvement in depth, contour, symmetry, and patency following Mercedes-pattern release, topical silver nitrate application, and foam earplug stenting (Image A). Silver nitrate reapplication (Image B) and foam earplug replacement with a new sterile unit (Image C) were performed at this visit, with a third and final application planned to occur one month later.

Topical silver nitrate was subsequently applied to suppress hypergranulation tissue, leveling the wound surface and promoting organized epithelialization across the smoothed tissue bed. A sterile foam earplug was introduced as a temporary conforming stent to maintain patency and prevent recurrent centripetal contraction during scar maturation. At one-month follow-up, the umbilicus demonstrated marked improvement in depth, contour, symmetry, and patency without recurrent stenosis. Silver nitrate was reapplied, the earplug replaced, and the patient instructed to return one month later for a third and final application (Figure 4). 

**Figure 5:**
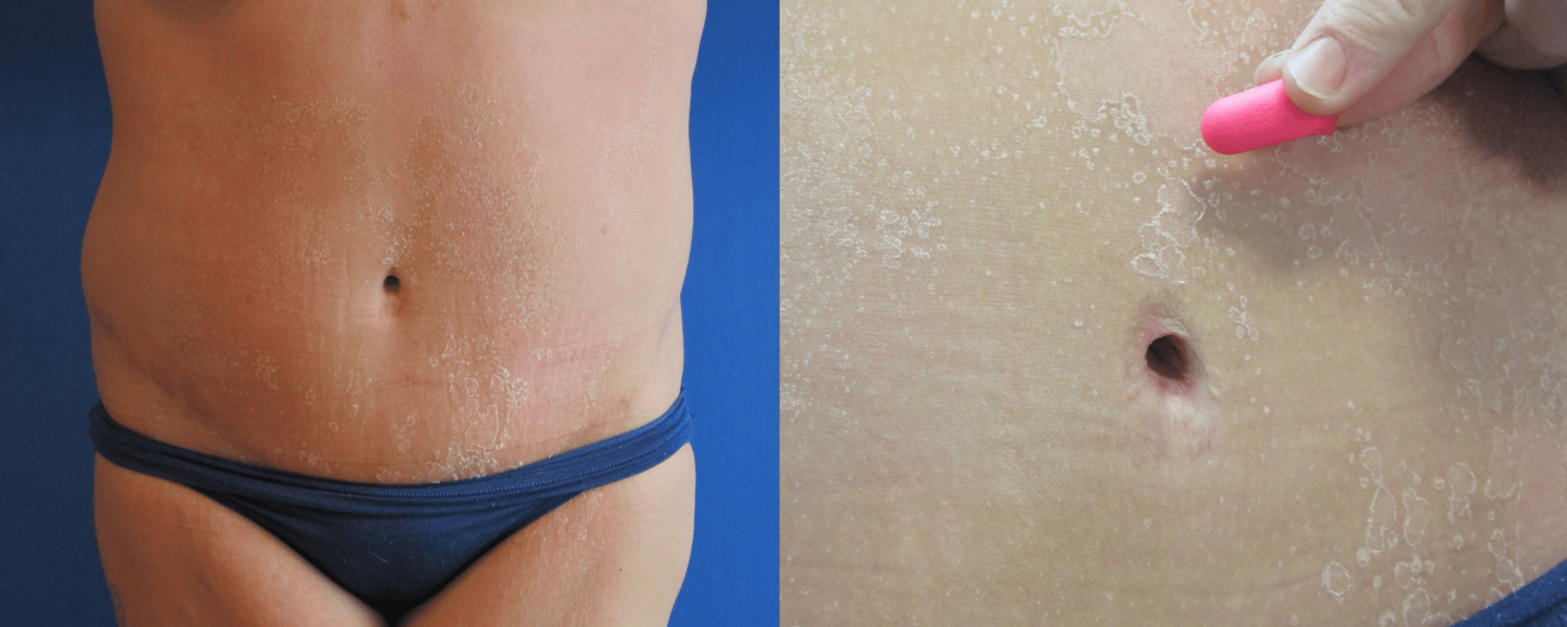
Reconstructed umbilicus Final reconstructive outcome of the umbilicus achieved through the Mercedes-pattern release technique, silver nitrate application, and foam earplug stenting.

At three months, the umbilicus maintained appropriate aperture and a satisfactory aesthetic profile (Figure 5).

## Discussion

Postoperative umbilical stenosis results from cicatricial contracture and progressive tissue remodeling inherent to wound healing, and occurs in up to 4.5% of patients post-abdominoplasty [8-10]. Myofibroblast proliferation and extracellular matrix remodeling generate centripetal forces on periumbilical tissues, representing the primary mechanism of contracture [9]. The umbilical ring and its attachment to the rectus sheath contain few elastic fibers, rendering it particularly susceptible to scar-driven contraction [11]. As scar tissue forms around the fibrous ring, the umbilical aperture narrows and loses patency, as seen in other circular incisions throughout the body [12,13]. When left unaddressed, this centripetal process can progress to near-complete obliteration, reducing any circular aperture to a pinpoint opening.

Vascular compromise represents an additional contributing factor. Circumferential freeing of the umbilicus during abdominoplasty leaves the stalk as the sole perfusion source. Excessive tension, torsion, or stalk ischemia can lead to tissue atrophy, fibrosis, and further centripetal contraction [12,14,15]. The degree of shrinkage varies with wound healing capacity, periumbilical defatting, stalk tension, and integrity of the deep inferior epigastric arteries and subdermal plexus [16]. 

The aesthetic consequences of umbilical stenosis are significant. As the central focal point of the abdomen, umbilical appearance heavily influences the overall perception of surgical success. A misshapen or malpositioned navel can undermine an otherwise well-performed procedure and contribute to psychological distress in patients who perceive their result as unnatural [17]. While reported rates of umbilical dissatisfaction following abdominoplasty vary considerably across the literature, figures as high as 25% have been cited, underscoring the aesthetic importance of umbilical preservation and the clinical relevance of managing its complications [6]. In addition to aesthetic significance, a significantly narrowed aperture may impair hygiene and limit adequate drying of the umbilical cavity, predisposing to moisture retention, skin maceration, and possible subsequent omphalitis.

Management of umbilical stenosis spans a spectrum from non-flap techniques, including stenting and purse-string sutures, to local flap reconstruction and, in cases of vascular compromise, pedicled perforator-based flaps. Non-flap approaches risk recurrent stenosis by failing to address the underlying cicatricial wound-healing response, while flap-based reconstruction carries greater operative complexity, donor site morbidity, and variable long-term aesthetic outcomes. The breadth of described techniques reflects the absence of a standardized approach.

In our experience, combining a Mercedes-pattern incision with topical silver nitrate and foam earplug stenting produced an uncomplicated, aesthetically pleasing result. In this technique, the Mercedes-pattern incision converts the contracted circular scar into three small flaps, releasing circumferential tension while preserving and redistributing native umbilical tissue. Silver nitrate complements this mechanism by releasing free silver ions that chemically cauterize periumbilical tissue, prevent excessive granulation, exert antimicrobial activity, and attenuate inflammatory fibroblast proliferation, promoting organized epithelialization and limiting progressive fibrosis [18]. As the scar matures, foam earplug stenting maintains aperture patency. Together, this combined approach addresses both the mechanical and biological drivers of stenosis. Importantly, this simple technique can be performed entirely in an outpatient or office-based setting at minimal cost and with negligible operative burden while still achieving a satisfactory outcome--an important consideration given the variable accessibility and morbidity associated with the alternative approaches described above. To our knowledge, this report is the first to describe the use of topical silver nitrate in the management of umbilical stenosis, offering a biologically targeted adjunct to existing techniques that directly counteracts the wound-healing mechanisms underlying progressive contracture.

This report is limited by its single-patient design, relatively short follow-up duration, lack of objective scar assessment scales, and the absence of patient-reported satisfaction measures, which were not collected as part of this case.

## Conclusions

The combination of Mercedes-pattern scar release, topical silver nitrate, and temporary foam earplug stenting provides a simple, reproducible, low-cost, and low-morbidity approach to preserving umbilical patency and aesthetics following abdominoplasty. Silver nitrate’s dual antiseptic and hemostatic properties support organized wound healing while attenuating the cicatricial response driving progressive contracture. Given its minimal operative burden and favorable cosmetic outcomes, this technique represents a valuable addition to the surgical armamentarium for the management of postoperative umbilical stenosis.
